# Identification, Characterization and Expression Analysis of TRP Channel Genes in the Vegetable Pest, *Pieris rapae*

**DOI:** 10.3390/insects11030192

**Published:** 2020-03-18

**Authors:** Fen Mao, Wan-jun Lu, Yi Yang, Xiaomu Qiao, Gong-yin Ye, Jia Huang

**Affiliations:** Ministry of Agricultural and Rural Affairs Key Laboratory of Molecular Biology of Crop Pathogens and Insects, Institute of Insect Sciences, Zhejiang University, Hangzhou 310058, China; maofenmaofen@163.com (F.M.); 21816189@zju.edu.cn (W.-j.L.); yylqy@zju.edu.cn (Y.Y.); xmqiao@zju.edu.cn (X.Q.); chu@zju.edu.cn (G.-y.Y.)

**Keywords:** *Pieris rapae*, transient receptor potential channel, splice isoform, gene duplication

## Abstract

Transient receptor potential (TRP) channels are critical for insects to detect environmental stimuli and regulate homeostasis. Moreover, this superfamily has become potential molecular targets for insecticides or repellents. *Pieris rapae* is one of the most common and widely spread pests of Brassicaceae plants. Therefore, it is necessary to study TRP channels (TRPs) in *P. rapae*. In this study, we identified 14 TRPs in *P. rapae*, including two *Water witch* (*Wtrw*) genes. By contrast, only one *Wtrw* gene exists in *Drosophil*a and functions in hygrosensation. We also found splice isoforms of *Pyrexia* (*Pyx*), *TRPgamma* (*TRPγ*) and *TRP-Melastatin* (*TRPM*). These three genes are related to temperature and gravity sensation, fine motor control, homeostasis regulation of Mg^2+^ and Zn^2+^ in *Drosophila*, respectively. Evolutionary analysis showed that the TRPs of *P. rapae* were well clustered into their own subfamilies. Real-time quantitative PCR (qPCR) showed that PrTRPs were widely distributed in the external sensory organs, including antennae, mouthparts, legs, wings and in the internal physiological organs, including brains, fat bodies, guts, Malpighian tubules, ovaries, as well as testis. Our study established a solid foundation for functional studies of TRP channels in *P. rapae*, and would be benefit to developing new approaches to control *P. rapae* targeting these important ion channels.

## 1. Introduction

The transient receptor potential (TRP) family members are six-transmembrane domain cationic channels with remarkable functions, such as thermosensation, chemosensation (smell and taste), vision, mechanosensation (hearing and touch), hygrosensation and others to perceive the external environment [1,2]. In 1969, the first *trp* gene was identified from a *Drosophila* mutant with defective vision [3]. The mutant flies showed electroretinogram response to light transiently, therefore this super family is named the transient receptor potential (TRP) [4]. After half a century development, TRP genes have been found and studied a lot in eukaryotes. It is divided into groups 1 and 2 based on sequence and topological differences [1]. The groups 1 TRPs include five subfamilies, TRP-Canonical (TRPC), TRP-Vanilloid (TRPV), TRP-Melastati (TRPM),TRP-Ankyrin (TRPA) and TRP-No mechanoreceptor potential C (TRPN) respectively, which share higher sequence similarity with *trp* gene than groups 2 TRPs. In *Drosophila*, 11 genes were identified belonging to these five subfamilies. Complete TRPC proteins usually contain 3–4 ankyrin repeats and can be activated by phospholipase C (PLC) [5]. This subfamily consists of 3 members, TRP, TRP-Like (TRPL) and TRPgamma (TRPγ). TRPV proteins usually contain 3–5 ankyrin repeats and can be activated by the inflammatory vanilloid compound capsaicin [6]. This subfamily of *Drosophila* includes Inactive (Iav) and Nanchung (Nan). TRPM is the only subfamily of group 1 TRPs without ankyrin repeats in *Drosophila*. It has only one member, named TRPM. A significant structural feature of TRPA subfamily is that large numbers of ankyrin repeats are located in the N-terminal domain (8–18) [7]. The TRPA subfamily has four members in *Drosophila*, named TRPA1, Painless (Pain), Pyrexia (Pyx) and Water witch (Wtrw). However, the TRPN family has the most ankyrin repeats than any other TRPs [8]. No mechano-receptor potential C (NompC) is the single member of *Drosophila* TRPN. Compared with the large group 1, group 2 has only two subfamilies, TRP-Polycystin (TRPP) and TRP-Mucolipin (TRPML), which are more distant from trp gene and have no ankyrin repeats [9]. *Drosophila* TRPP subfamily includes Polycystin-2 (Pkd2), Brivido1–3 (Brv1–3). Brivido proteins contain 8–10 transmembrane segments rather than 6 [10]. The other subfamily TRPML has only one member, named TRPML.

The small cabbage white butterfly, *Pieris rapa*e (Lepidoptera: Pieridae), known as imported cabbageworm or European cabbage butterfly, is one of the most common and widely spread pests of Brassicaceae plants [11]. *P. rapae* larvae eat vegetable leaves reducing cabbage plants to stems [12]. In order to reduce the loss of production, chemical control methods as the most convenient to spread in large area are still widely used in agricultural production. While as the long-term use of a few chemical pesticides, the *P. rapae* has produced resistances [13]. Therefore, it is necessary to develop alternative agents.

In recent years, TRP channels have gradually emerged as the molecular target of noxious chemicals. A previous study showed that *Drosophila* TRPA1 channel is required to avoid the insect repellent citronellal [14]. *Painless* mutant flies are defective in avoidance of isothiocyanate (ITC), the pungent ingredient of wasabi [15]. TRPA1 of *Anopheles gambiae* are directly activated by citronellal and benzaldehyde [14]. The commercial insecticides pymetrozine, pyrifluquinazon and afidopyropen directly act Nan-Iav complexes specifically expressed in chordotonal neurons, causing insects uncoordinated and eventually dead [16]. In general, TRP channels have become potential molecular targets for insecticides or repellents [17]. Thus, it is meaningful to identify the sequences and characteristics of TRP channels in important agricultural or hygienic insects.

In this study, we identified 14 TRP genes in *P. rapae* and investigated their spliced isoforms and tissue distributions. Supplementary identification of TRP genes was also done in three other lepidoptera insects (the monarch butterfly *Danaus plexippus*, the Asian swallowtail *Papilio Xuthus* and the tobacco hornworm *Manduca sexta*) and the brown planthopper *Nilaparvata lugens* (Stål) (Hemiptera: Delphacidae), one of the most important rice pests in temperate and tropical regions; the damp-wood termite *Zootermopsis nevadensis* (Isoptera: Archotermopsidae), one common pest in temperate forests; and the German cockroach *Blattella germanica* (Blattodea: Blattellidae), one notorious sanitary pest. 

## 2. Materials and Methods 

### 2.1. Insect

The colonies of *P. rapae* were collected from cabbage fields in Hangzhou, Zhejiang province, China and were all reared continuously on cabbage leaves in the temperature-controlled room (25 ± 1°C, 70%–80% relative humidity (R.H.), and a photoperiod of 14:10 (L:D) h with the light intensity of 3500–4000 Lux). Adult *P. rapae* were fed with 10% sucrose water containing 0.15% riboflavin for several days.

### 2.2. Identification of TRP Channels

First of all, to search TRP genes in each species, we used TRP protein sequences of *Drosophila* to blast transcriptomic or proteomic data released in National Center for Biotechnology Information (NCBI) (https://www.ncbi.nlm.nih.gov/). If any genes were not found, we would go to blast the genome database for supplementing or confirming our results. Then, candidate genes were further verified using BLASTP versus the Database of *Drosophila* Genes and Genomes (FlyBase) (http://flybase.org/). One exception is TRPA5, because *Drosophila* does not have TRPA5. As long as the candidate genes were not Pyx orthologues, we considered that sequences as TRPA5.

### 2.3. Total RNA Isolation, Reverse Transcription and Splice Variants Detection

Total RNAs from fifth-instar *P. rapae* larvae were extracted using the TRIzol (Invitrogen, Carlsbad, CA, US) according to the manufacturer’s instructions. Single-strand complementary DNA (cDNA), synthesized from 1000ng of total RNA using the TransScript First-Strand cDNA Synthesis SuperMix (TransGen Biotech, Beijing, China), were as then used as templates for PCR reactions. The PCR primers for investigating the splice variants were designed with primer5 (Table S1) [18]. PCR products were separated by electrophoresis on 1% agarose gel. The gel where target fragments located were cut off and DNA were purified with Agarose Gel DNA Extraction Kit-250 prep (Esay-Do, Hangzhou, China). Then the purified PCR products were cloned into pEASY-Blunt Zero (Transgen, Beijing, China) and transformed into DH5α competent *E. coli* cells (Vazyme, Nanjing, China). Positive clones were sequenced by second-generation sequencing (Sunya, Hangzhou, China).

### 2.4. Bioinformatic Analysis

Sequence similarity for orthologous genes was performed using BLASTP program on the National Center for Biotechnology Information (NCBI). Protein domains were predicted by TMHMM 2.0 (http://www.cbs.dtu.dk/services/TMHMM-2.0/) and SMART (http://smart.embl-heidelberg.de/). Sequences were aligned by ClustalW2 (http://www.ebi.ac.uk/Tools/msa/clustalw2/). The amino acid sequences in phylogenetic tree were aligned through MAFFT software v7.123b with the default parameters [19], and then IQ-TREE was applied for the maximum likelihood analyses [20], under the LG + G4 nucleotide substitution model predicted by ModelFinder [21] and with 1000 bootstrap replicates for testing the credibility of evolutionary tree [22].

### 2.5. Real-Time Quantitative PCR and Statistical Analysis

Total RNAs from antennae, compound eyes (with optic lobes), mouthparts, legs, wings, brain (without optic lobes), malpighian tubules, gut and fat bodies as well as ovaries and testes of about 25 adults of *P. rapae* (3–5 days old) were extracted using the TRIzol (Invitrogen, Carlsbad, CA, US) according to the manufacturer’s instructions. The concentrations and quantities of RNA were measured by using a Nanodrop 2000 spectrophotometer (Thermo Scientific Inc., Bremen, Germany). Then 1ug RNA was used to perform reverse transcription reaction in each sample. A total of 10×cDNA was used as a template for real-time quantitative PCR (qPCR). qPCR reactions were performed using CFX96™ Real-Time PCR Detection System (Bio-rad, Hercules, CA, USA) with ChamQ^TM^ SYBR qPCR Master Mix (Without ROX) (Vazyme, Nanjing, China) according to the manufacturer’s instructions. Three sample biological replicates and two mechanical repeats were carried out in each sample. Sequence-specific primers for qPCR were designed using AlleleID 6 (Table S2) [23]. cDNAs were normalized by amplification of 18s rRNA [24]. Quantification of transcript level was quantified using the 2^-ΔΔCT^ method [25]. One-way analysis of variance (ANOVA) and Tukey’s multiple comparison test were employed to analyze the data of relative expression levels in various tissues. For the comparison of expression levels between splice variants and gene duplications, unpaired t test was applied. The analysis software was Statistical Product and Service Solutions (SPASS).

## 3. Results

### 3.1. Identification and Phylogenetic Analysis of TRP Channels in P. Rapae and Other Lepidoptera Insects

**Figure 1:**
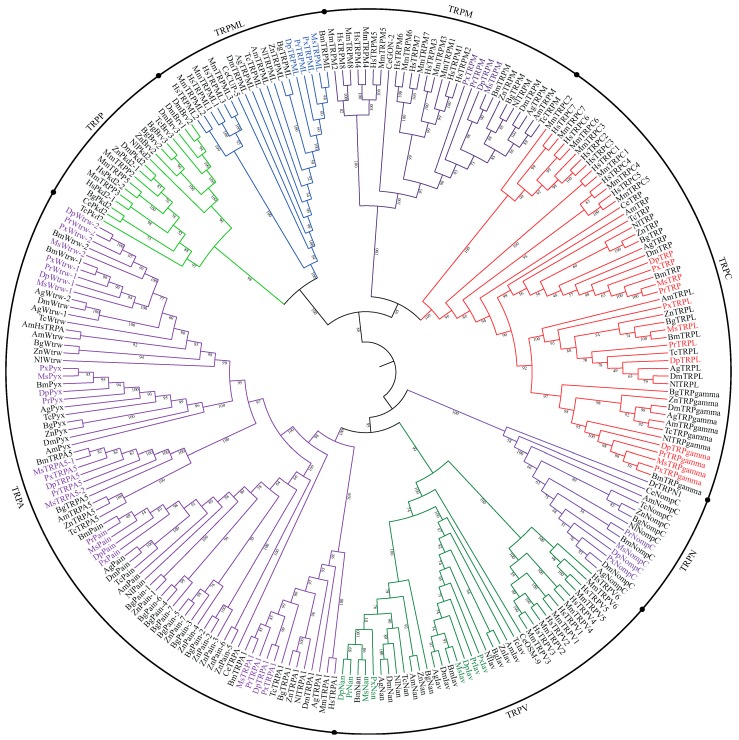
Phylogenetic analysis of TRP channels in *P. rapae*, *D. plexippus*, *P. Xuthus*, *M. sexta*, *B. mori* and other organisms. Amino acid sequences of *P. rapae* (Pr), *D. plexippus* (Dp), *P. Xuthus* (Px), *M. sexta* (Ms), *B. mori* (Bm), *B. germanica* (Bg), *Z. nevadensis* (Zn), *N. lugens* (Nl), *Apis mellifera* (Am), *D. melanogaster* (Dm), *A. gambiae* (Ag), *Tribolium castaneum* (Tc), *Caenorhabditis elegans* (Ce), *Homo sapiens* (Hs), *Danio rerio* (Dr) and *Mus musculus* (Mm) TRP channels were aligned through MAFFT software v7.123b with the default parameters [18]. Under the LG + G4 nucleotide substitution model, IQ-TREE was applied for the maximum likelihood analyses with 1000 bootstrap replicates [19]. The bootstrap value is indicated next to each branch. The accession number of TRP channels identified in this study are listed in an Additional file (Table S3). Other accession numbers of identified TRP channels originated from previous studies [26,27].

We identified 14 TRP channel genes in *P. rapae*, the monarch butterfly *Danaus plexippus*, the Asian swallowtail *Papilio Xuthus*, and 15 TRP channel genes in the tobacco hornworm *Manduca sexta* (Table 1). Phylogenetic analysis arranged these sequences into TRPC, TRPA, TRPN, TRPV, TRPM and TRML, but not into TRPP (Figure 1). Compared with other insects, *Pkd2*, *Brv* and *Hymenoptera-specific TRPA* (*HsTRPA*) were not found in the above four Lepidoptera insects as well as the silkworm *Bombyx mori* (Table 2). However, *TRPA5* were found in their genomes and two *TRPA5* existed in *M. sexta*. In addition, all five lepidoptera insects had two *Wtrw* genes. They were well divided into two classes in evolutionary analysis (Figure 1).

### 3.2. Sequence Analysis, Splice Variants and Gene Duplications of TRP Channelsin P. Rapae

We did a detailed sequence analysis of TRP channels in *P. rapae*. A blastp program revealed that most TRPs in *P. rapae* shared a high sequence identity (above 50%) with TRPs in *D. melanogaster* (Table 1). Only PrPain and PrWtrw, 2 of TRPA subfamily shared under 50% sequence identity with their homologous sequence in *D. melanogaster*, which may be due to the rapid evolution of TRPA subfamily in insects [28]. All other *P. rapae* TRPs had six transmembrane domains except that PrNan had only four transmembrane domains for its incomplete sequence. Consistent with other species [7], TRPN of *P. rapae* had the most N-terminal ankyrin repeats domain, followed by TRPA, TRPV and TRPC, TRPM and TRPML still have no ankyrin repeats (Table 1).

**Figure 2:**
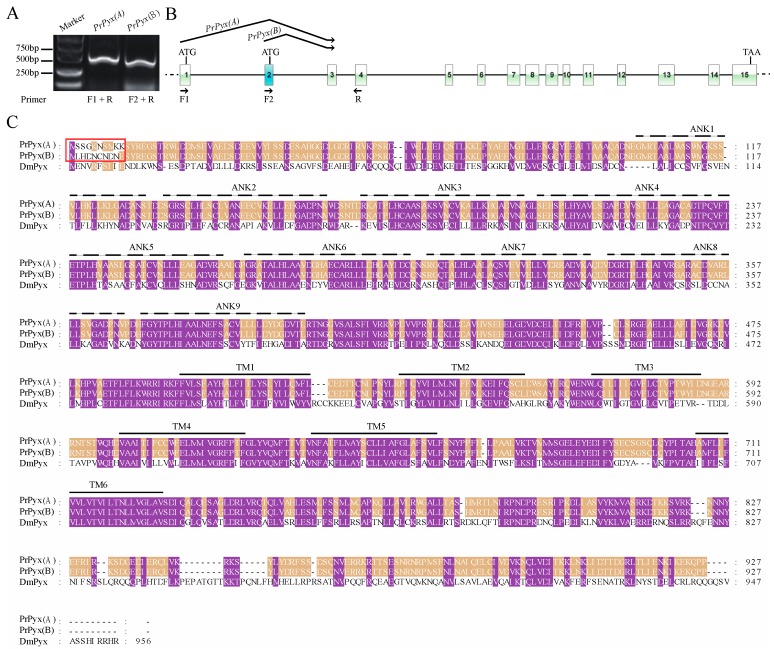
Detection and amino acid sequence alignment of the two *PrPyx* splice forms. (**A**) Two molecular isoforms of *PrPyx* mRNAs identified by PCR. (**B**) Two-dimensional gene structure map of *PrPyx*. The boxes represented the exons of *PrPyx*, the black solid lines connecting the boxes represented the introns, and the black dotted lines on both sides represent other genome sequences. (**C**) Multiple sequence alignment deduced for PrPyx(A), PrPyx(B) and DmPyx. The red box indicates the different amino acid residues of the two isoforms. Ankyrin repeats were indicated with dotted lines and marked as ANK1–9. Transmembrane regions were indicated with solid lines and marked as TM1–6. The protein sequences used for multiple sequence alignment were in the attached fasta file.

**Figure 3:**
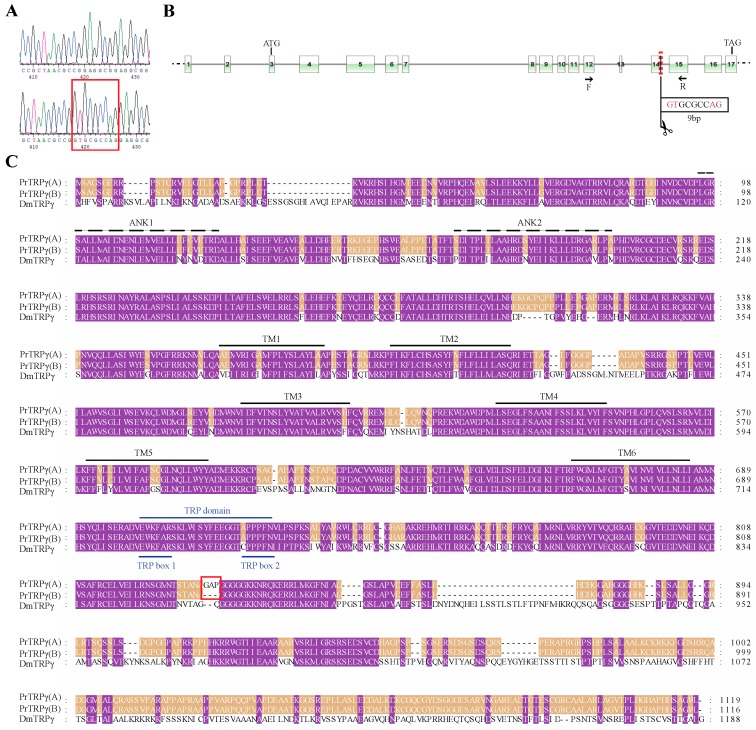
Detection and amino acid sequence alignment of the two *PrTRPγ* splice forms. (**A**) Two molecular isoforms of *PrTRPγ* mRNAs identified by PCR. (B) Two-dimensional gene structure map of *PrTRPγ*. The boxes represented the exons of *PrTRPγ*, the black solid lines connecting the boxes represented the introns and the black dotted lines on both sides represent other genome sequences. The deletion of 9 nucleotides at the end of exon 14 produced by splicing were showed in the black box. (**C**) Multiple sequence alignment deduced for PrTRPγ(A), PrTRPγ(**B**) and DmTRPγ. The red box indicates the different amino acid residues of the two isoforms. TRP boxes and TRP domain were indicated with blue lines. Ankyrin repeats were indicated with dotted lines and marked as ANK1–2. Transmembrane regions were indicated with solid lines and marked as TM1–6. The protein sequences used for multiple sequence alignment were in the attached fasta file.

**Figure 4:**
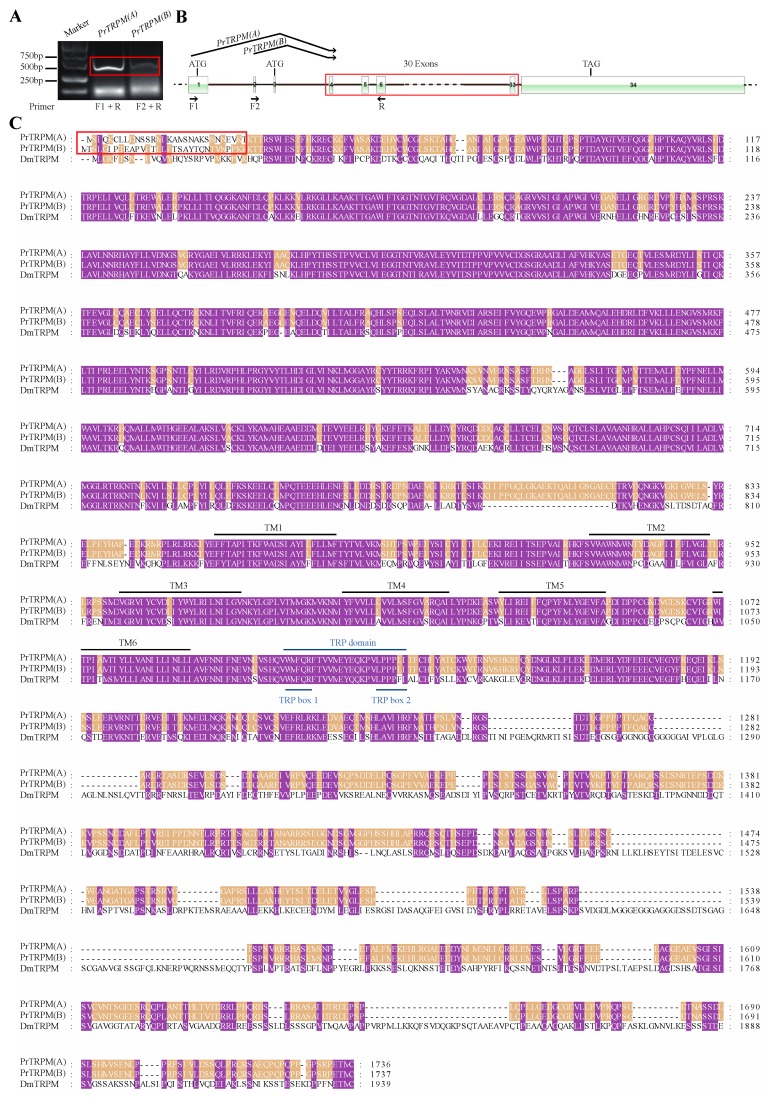
Detection and amino acid sequence alignment of the two *PrPRPM* splice forms. (**A**) Two molecular isoforms of *PrTRPM* mRNAs identified by PCR. The bands of *PrTRPM* mRNAs were indicated with open red box, and the bands below it were primer dimers or none-specific bands. (**B**) Two-dimensional gene structure map of *PrTRPM*. The boxes represented the exons of *PrTRPM*, the black solid lines connecting the boxes represented the introns and the black dotted lines on both sides represent other genome sequences. (**C**) Multiple sequence alignment deduced for PrTRPM(A), PrTRPM(B) and DmTRPM. The red box indicates the different amino acid residues of the two isoforms. TRP boxes and TRP domain were indicated with blue lines. Ankyrin repeats were indicated with dotted lines and marked as ANK1–2. Transmembrane regions were indicated with solid lines and marked as TM1–6. The protein sequences used for multiple sequence alignment were in the attached fasta file.

After we found two splice variants for *PrPyx*, we mapped the splice variants to the genomic sequence for checking the GA-AG rule about introns. Then we performed PCR on cDNA of *P. rapae* larvae using specific primers and sequenced the PCR products (Figure 2A). Results of these two steps indicated that the two splice isoforms both consisted of 14 exons which included the first two alternative and mutually exclusive exons and the last 13 shared exons (Figure 2B). Their open reading frames (ORFs) both encoded 928 amino acids, but there were still 9 different amino acids at the 5 ‘end of protein sequences (Figure 2C). Even though, the two deduced protein sequences for PrPyx both had 9 N-terminal ankyrin repeats domains and 6 transmembrane domains. Then we verified two splice variants for *PrTRPγ* in the same way (Figure 3A). The difference between two transcripts originated from the deletion of 9 nucleotides at the ends of 14th exon (Figure 3B). Predictions of protein structure showed that the difference of variable splicing existed in TRP domain (Figure 3C), which indicated that this splicing maybe played an important role in physiological function. Ankyrin repeats domains and transmembrane domains had no difference between the two transcripts (Figure 3C). *PrTRPM* was also spliced into two isoforms (Figure 4A). By investigating genomic and transcriptome data, we found the exons of their initial codons were mutually exclusive (Figure 4B). Therefore, there were about 30 N-terminal amino-acid residues differences in their predicted protein sequences (Figure 4C).

**Figure 5:**
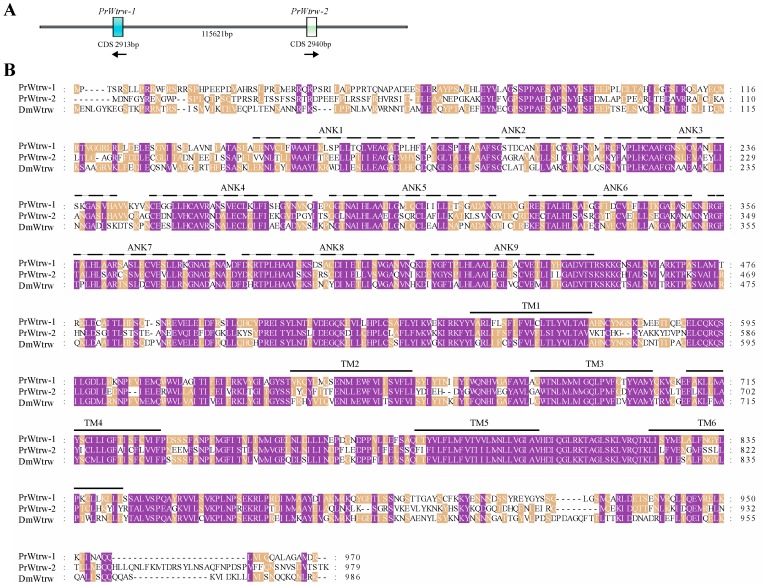
Sequence analysis of two *PrWtrw* genes. (**A**) Duplication diagram of the *PrWtrw* gene. Length of two coding sequences (CDSes) and interval length of two genes were shown as the figure. Arrows showed the direction of gene transcription. (**B**) Multiple sequence alignment deduced for PrWtrw-1, PrWtrw-2 and DmWtrw. Ankyrin repeats were indicated with dotted lines and marked as ANK1–2. Transmembrane regions were indicated with solid lines and marked as TM1–6. The protein sequences used for multiple sequence alignment were in the attached fasta file.

As mentioned above, gene duplications of *Wtrw* were found in all five lepidoptera insects (Table 1). Two *Wtrw* genes of *P. rapae* existed in the same scaffold with the reverse direction of transcription (Figure 5A). The results of functional domain prediction showed that PrWtrw-1/2 had the same transmembrane domains and different ankyrin repeats domains. In addition to the shared last eight ankyrin repeats, PrWtrw-1 had another unique ankyrin repeat in front of its N-terminal ankyrin repeats domain (Figure 5B).

### 3.3. Tissue Expression Profiles of TRP Channel in Adult P. Rapae

**Figure 6:**
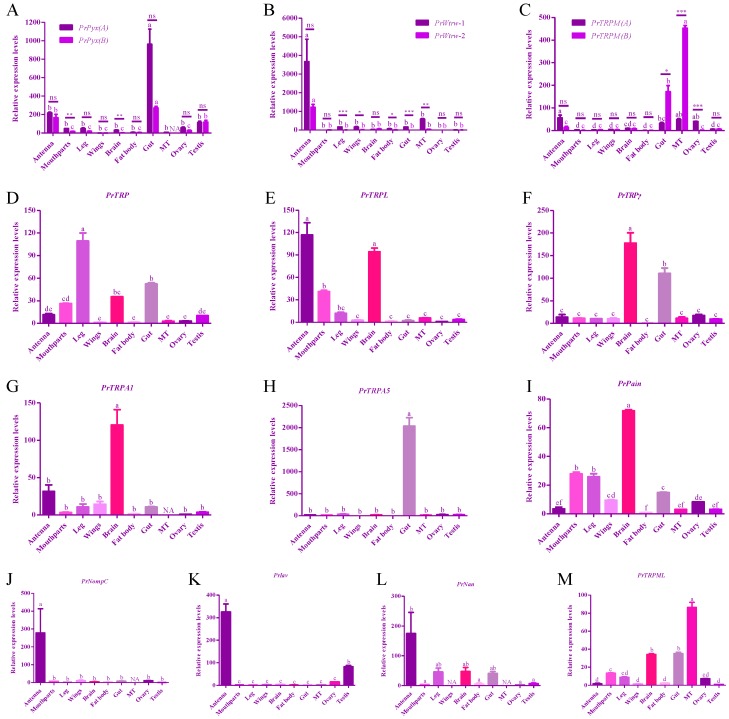
Relative transcriptional levels of two isoforms of *PrPyx* (**A**), *PrWtrw* (**B**), *PrTRPM* (**C**), *PrTRP* (**D**), *PrTRPL* (**E**), *PrTRPγ* (**F**), *PrTRPA1* (**G**), *PrTRPA5* (**H**), *PrPain* (**I**), *PrNompC* (**J**), *PrIav* (**K**), *PrNan* (**L**) and *PrTRPM**L*** (**M**) in various tissues of 3–5 days old adult *P. rapae.* “MT” represented malpighian tubules. Asterisks showed the significant difference in expression levels of two isoforms and gene duplications in the same samples. Data represent means  ±  SEM.

Insects have to constantly sense the environmental stimuli and adjust homeostasis to ensure their normal life activities. TRP channel functions not only in sensory physiology, but also in homeostasis [2]. Thus, in order to investigate the possible roles of TRPs in *P. rapae*, we evaluated the relative transcription levels of TRPs in the external sensory organs, including antennae, mouthparts, legs, wings, and in the internal physiological organs, including brains, fat bodies, guts, Malpighian tubules, ovaries, testis. Results showed that transcription levels of PrTRP were different in various tissues, and the expression level in legs was the highest, while that in wings, fat bodies, Malpighian tubes and ovaries were low (Figure 6A). *PrTRPL* was mainly transcribed in antenna and brains (Figure 6B). Abundant transcripts of *PrTRPγ* were detected not only in brains but also in guts (Figure 6C). *PrTRPA1* was highly expressed in the brains, but it is rarely detected in the Malpighian tubes (Figure 6D). Compared with the high transcription level of *PrTRPA5* in guts, the transcription levels in other tissues were very low and had no significant difference (Figure 6E). Like *PrTRPA5*, the highest messenger RNA (mRNA) levels of *PrPyx* were observed in guts (Figure 6F). mRNA levels of *PrPyx(A)* in antenna and testis were significantly higher than that of other tissues (Figure 6F). In mouthparts and brains, mRNA levels of *PrPyx* (primer-amplified both *PrPyx(A)* and *PrPyx(B)* were significantly higher than that of *PrPyx(A),* indicating that *PrPyx(B)* may be the main form in these two tissues of adult *P. rapae* (Figure 6F). The highest expression levels of both *PrWtrw-1* and *PrWtrw-2* were detected in antenna (Figure 6G). Higher transcription levels of *PrWtrw-1* than *PrWtrw*-*2* were found in legs, wings, brains, guts and Malpighian tubules (Figure 6G). Transcription levels of *PrPain* were higher in brains, mouthparts and legs than in other tissues (Figure 6H). *PrNompC*, *PrIav* and *PrNan* were all detected the highest mRNA levels in antennae (Figure 6I-K). In addition, *PrIav* was highly transcribed in testis, and *PrNan* was highly transcribed in legs, brains and guts (Figure 6J-K). *TRPM(A)* had higher mRNA levels in antenna, guts, Malpighian tubules and ovaries, while *TRPM(B)* only had higher mRNA levels in guts and Malpighian tubules (Figure 6L). *TRPM(B)* mRNA was significantly more than *TRPM(A)* mRNA in guts and Malpighian tubules, and was significantly fewer that *TRPM(B)* mRNA in ovaries (Figure 6L). *TRPML* had the highest mRNA level in Malpighian tubules, followed by brains and guts (Figure 6M).

## 4. Discussion

In this study, we identified 14 TRPs in *P. rapae*, *D. plexippus*, *P. Xuthus*, and 15 TRPs in *M. sexta* that be divided into TRPC, TRPA, TRPN, TRPV, TRPM and TRML, but not into TRPP based on their structure and phylogenetic analyses. As the most ancient TRP subfamily, TRPP channels are gated by Ca^2+^ flux [29]. *Drosophila* TRPP involves in not only larval feeding behavior, but also in directional sperm movement [30,31]. However, TRPP were absent in the five lepidoptera species we investigated. It indicated the functions of TRPP may be compensated by other TRPs or alternative pathways in lepidoptera insects. In addition, we identified 3 TRPPs in *Z. nevadensis*, 2 TRPPs in *B. germanica* and 1 TRPPs in *N. lugens* (Table 2), suggesting that the numbers and functions of TRPPs varied among different insects. Compared with only 1 TRPA channel in mammals, the member of this subfamily was highly diverse in arthropod species, especially insects (Table 2) [26]. As shown above, although TRPA5 was absent in *Drosophila* and *N. lugens*, it was present in all five lepidoptera insects, *Z. nevadensis* and *B. germanica* (Table 2). However, the physiological roles and gating mechanism of TRPA5 channel had yet to be determined in any organism. Previous researches have shown the lack of TRPA1 probably can be compensated by HsTRPA [28,32]. TRPA1 was present in all eight insects we investigated, which may explain why HsTRPA was absent in their genomes. In our study, the duplication of *Wtrw* was only found in Lepidoptera insects, which may be due to their specific habitats and life histories. So far, studies on the functions of *Wtrw* have been limited in *Drosophila*, duplications of *Wtrw* in Lepidoptera will provide more perspectives for its physiological roles. We identified more than one Pain channels in *Z. nevadensis* and *B. germanica*. While only ZnPain-1 and BgPain-1 were clustered together with Pain channels of other ten insects, and their branch evolved later (Figure 1). It implied various physiological functions which required multiple Pain channels in ancient insects could be undertaken by only one Pain channel through evolution. The functions of disappeared Pyx in *N. lugens* may be compensated by other TRPs or alternative pathways.

Studies in *Drosophila* have shown that TRPC subfamily expressed in photoreceptors and the brain participate in phototransduction [9,33]. In this study, we failed to get clean samples of compound eyes for quantitative expression analysis, while we found high expression of TRPCs in the brains of *P. rapae*. Many bristles on leg, mouthparts and antennae of insects sense various stimuli all the time, so the highly transcribed PrTRP and PrTRPL in these three tissues may play a role similar to the mammalian TRPC sensing ever-changing environmental conditions in the primary cilia [34]. In mammal, TRPC channels function in intestinal fibrogenesis [35], which may explain the relatively high transcription levels of *PrTRP* and *PrTRP*γ, the homologs of *TRPC*, in *P. rapae* guts. *Drosophila* TRPA1 channel is expressed in the AC (anterior cell) neurons that are located in the brain for temperature sensation [36], and robust transcripts of PrTRPA1 in *P. rapae* brains were also observed. In addition, TRPA1 is involved in other sensory processes, such as the avoidance of aversive odorants, non-volatile irritants and mechanical stimuli [2,14]. Most sensory organs are located in insect antennae, legs, mouthparts and wings [2], so we successfully detected mRNAs of PrTRPA1 in these tissues. In the case that the gating mechanism and function of TRPA5 are not clear, we found mRNA level of PrTRPA5 was significantly higher than that of other tissues, which may provide some basis for the function studies of TRPA5 channel. Pyx [26], the closest relative of TRPA5 (Figure 1), was also observed to have high transcription levels in *P. rapae* guts, implying that they may regulate the function of guts. *Drosophila* Pyx channel that expressed in the Johnston’s organ of antennae could regulate anti-gravitaxis behavior [37]. Accordingly, high mRNA level of *PrPyx(A)* was detected in antennae of *P. rapae*. Abundant transcripts of *PrPyx* in testis may affect reproductive behaviors. Consistent with previous reports, *Wtrw* and *NompC* were mainly transcribed in antennae, therefore they may be also related to hygrosensation and hearing respectively in *P. rapae* [38,39]. Pain was widely distributed in the external sensory organs and internal physiological organs. High mRNA levels of PrPain in the external sensory organs, such as legs, wings and mouthparts, suggested that PrPain was probably required for both thermal, chemical and mechanical nociception [40]. Abundant mRNA of PrPain in the internal physiological organs, such as brains, guts and ovaries, indicated that PrPain may had other extended roles in regulating various physiological processes. The TRPV channels Iav and Nan, another TRP channel expressed in insect antennae for hearing and gravity sensation [37,41], were also detected to have robust transcription in *P. rapae* antennae. Abundant mRNA of PrIav in testis may explain that mRNAs of Iav in male insects are higher than that in female [42]. This result also suggested that Iav may be involved in the reproductive physiology of male insects. Moderate expression levels of PrNan were detected in legs for the existence of peripheral neurons and brains for antennal neuron projections [39,43]. Moreover, the relatively abundant mRNA of PrNan in guts may be due to its roles similar to TRPV1 in mammalian gastrointestinal tract for secreting substances and protecting digestive tracts [44]. TRPM protein is required for Mg^2+^ and Zn^2+^ homeostasis and expressed in the Malpighian tubules to remove electrolytes and toxic components from the hemolymph [45,46]. Indeed, TRPM was transcribed at a high level in *P. rapae* Malpighian tubules as well as in guts, which indicated PrTRPM may not only regulate Mg^2+^/Zn^2+^ homeostasis but also maintain normal physiological functions of guts. Similar to the high transcription levels of TRPML3 in the intestine and kidney [47,48], abundant transcripts of *PrTRPML* were also detected in guts and Malpighian tubules of *P. rapae*, indicating its roles in digestion and excretion. Neurodegeneration and accumulation of late-apoptotic cells always occurred in the brain of *TRPML* mutant *Drosophila*, which may be the reason why abundant transcripts of *PrTRPML* were transcribed in the *P. rapae* brain.

## Figures and Tables

**Table 1:** Transient receptor potential (TRP) channels identified in *P. rapae.*

Subfamily	Gene Name	Genomic Sequence ID	NCBI Accession No. (Transcripts)	Length (Amino Acids)	Protein Region Identified	Number Of Ankyrin Repeats	CG No. Of The *Drosophila* *Melanogaster *Orthologue	Sequence Identity
**Group-1 TRPs**								
TRPC	*PrTRPγ*	NW_019093512.1	XM_022266966.1	1120	TM1–6	2	CG5996	65.81%
	*PrTRPL*	NW_019093246.1	XM_022259226.1	1158	TM1–6	2	CG18345	62.72%
	*PrTRP*	NW_019093246.1	XM_022259230.1	788	TM1–6	5	CG7875	51.81%
TRPA	*PrPain*	NW_019093434.1	XM_022264961.1	950	TM1–6	9	CG15860	36.86%
	*PrPyx*	NW_019093274.1	XM_022260547.1	928	TM1–6	9	CG17142	52.06%
	*PrTRPA1*	NW_019093159.1	XM_022269136.1	1134	TM1–6	14	CG5751	66.23%
	*PrWtrw-1*	NW_019093349.1	XM_022263017.1	971	TM1–6	9	CG31284	63.26%
	*PrWtrw-2*	NW_019093349.1	XM_022263000.1	980	TM1–6	8	CG31284	49.24%
	*PrTRPA5*	NW_019093774.1	XM_022269806.1	2350	TM1–6	16	-	-
TRPN	*PrNompC*	NW_019099827.1	XM_022272967.1	1607	TM1–6	29	CG11020	77.82%
TRPM	*PrTRPM*	NW_019099824.1	XM_022272780.1	1137	TM1–6	0	CG44240	61.22%
TRPV	*PrIav*	NW_019093844.1	XM_022270177.1	1061	TM1–6	5	CG4536	72.71%
	*PrNan*	NW_019093483.1	XM_022266197.1	584	TM1–4	5	CG5842	64.03%
**Group–2TRPs**								
TRPML	*PrTRPML*	NW_019093323.1	XM_022262542.1	600	TM1–6	0	CG8743	61.04%

**Table 2:** Number of TRP channels identified in *P. rapae*, *P. Xuthus*, *D. plexippus*, *M. sexta*, *Z. nevadensis*, *B. germanica*, *N. lugens* and previous reports [26,27].

Species Name	Channel type																
	TRPC			TRPA						TRPN	TRPV		TRPM	TRPP		TRPML	Total
	TRP	TRPL	TRPγ	TRPA1	TRPA5	HsTRPA	Pain	Pyx	Wtrw	NompC	Iav	Nan	TRPM	Brv	Pkd2	TRPML	
**Lepidoptera**																	
*B. mori*	1	1	1	1	1	0	1	1	2	1	1	1	1	0	0	1	14
*D. plexippus*	1	1	1	1	1	0	1	1	2	1	1	1	1	0	0	1	14
*M. sexta*	1	1	1	1	2	0	1	1	2	1	1	1	1	0	0	1	15
*P. Xuthus*	1	1	1	1	1	0	1	1	2	1	1	1	1	0	0	1	14
*P. rapae*	1	1	1	1	1	0	1	1	2	1	1	1	1	0	0	1	14
***Isoptera***																	
*Z. nevadensis*	1	1	1	1	1	0	7	1	1	1	1	1	1	1	2	1	22
**Blattodea**																	
*B. germanica*	1	1	1	1	2	0	7	1	1	1	1	1	1	0	2	1	22
**Hemiptera**																	
*N. lugens*	1	1	1	1	0	0	1	0	1	1	1	1	1	0	1	1	14
**Hymenoptera**																	
*A. mellifera*	1	1	1	0	2	1	1	1	1	1	1	1	1	0	0	1	13
**Diptera**																	
*A. gambiae*	1	1	1	1	0	0	1	1	1	1	1	1	1	0	0	1	13
*D. melanogaster*	1	1	1	1	0	0	1	1	1	1	1	1	1	3	1	1	16
**Coleoptera**																	
*T. castaneum*	1	1	1	1	0	0	1	0	1	1	1	1	1	0(1)	1	1	14 (15)

## Supplementary Materials

The supplementary materials are available at https://www.mdpi.com/2075-4450/11/3/192/s1. Table S1: Primers used for investigating the splice variants, Table S2: Primers used for qPCR, Table S3: Accession number of TRP channels identified in this study, Fasta file: The protein sequences used in multiple sequence alignments.
