# Supplementary material for: Identification, Characterization and Expression Analysis of TRP Channel Genes in the Vegetable Pest, Pieris rapae
Source: Insects. 2020 Mar 18;11(3):192. doi: 10.3390/insects11030192 (PMC7143563; doi:10.3390/insects11030192)
Supplement: Supplementary file 1 [file insects-11-00192-s001.pdf]

Table S1. Primers used for investigating the splice variants.

Primer name	Sequence (5'-3')	Length (bp)
PrPyx(A)-F	GAAATTTTTCTTCTGTGTCTTGCGG	487
PrPyx(B)-F	TAATCTGTTTATATTCGGTGGAGGC	468
PrPyx-R	CTTCAGCAACTTATGTAGCACGGAG	—
PrTRPM(A)-F	AAGCAAACCGAAACTTTCTTACGTC	569
PrTRPM(B)-F	AGTCTTATTTTACGTGTTCCCGCAT	512
PrTRPM-R	AACGCCCATTCTCGTGTAAGTAGTT	—
PrTRPγ-F	AATTGTGGATCAGCTATTTCGAAGA	689
PrTRPγ-R	GACCGATGAGACGTGAAACTCG	

Table S2. Primers used for qPCR.

Primer name	Sequence (5'-3')
qPrIav-F	CGCCAGTTCGCTTCATCTTGC
qPrIav-R	GTGGTATTCGCTGTTGGTCTCTTG
qPrNan-F	AGGAGCGTGCGAGACATAAGC
qPrNan-R	TCAGCAGGCAGAGATGAAGGATTG
qPrNompC-F	GTGCTCATAACGCTCCTCATTGC
qPrNompC-R	CGCTCATCTGGTCTTGTCTCTGG
qPrPain-F	CGGGATCAAAGAGCCATCAAATCG
qPrPain-R	TGCGTAGTGTAATGCTGTGTTGTC
qPrPYX(A+B)-F	CGCCGAGGATTCTGATGAGGAG
qPrPYX(A+B)-R	TTCATTGTCTGCTTGTGCTGCTG
qPrTRPA1-F	CACGAGCAAAGAGCCCAAGAAG
qPrTRPA1-R	GGAGGACACAGAGCAGAGAAGG
qPrTRPγ(A+B)-F	CGGTTCTCTGTCGGCTCTACTC
qPrTRPγ(A+B)-R	TGTGGTGCGGTGGCTTGC
qPrTRPL-F	CACAAGCATCCAGCACCATATAGC
qPrTRPL-R	CATTCATCGCAGCCGCATCG
qPrTRPM(A)-F	TGCGTGGTGCTGAGGAAGATG
qPrTRPM(A)-R	ATGCGTGGTGTTAGCGAGAGG
qPrTRPM(B)-F	CGTCACTGGGTATACCTACT
qPrTRPM(B)-R	CTTGCTTAGGCCACACCAAC
qPrTRPML-F	ACGGACATACACCAACTTGAGGAG
qPrTRPML-R	TTACGCTTTGCTCGCCATTTCTC
QPrWtrw-1-F	CGTAAACTGCGAAGGCGGATTG
QPrWtrw-1-R	GCGGCTAAGTGTAATGCTGTAGAC
QPrWtrw-2-F	CCTGCTGAAGATGCTCCGAATATG
QPrWtrw-2-R	ATCTCCCTGCTCCCAGAGTTAAAG
qPrTRPA5-F	GATAACATCGCAACTCCGCTACAC
qPrTRPA5-R	GGGCACCCAGGCTTCTTCG
qPrTRP-F	TGAACCCGAATCACCATCCA
qPrTRP-S	CCTTTGCTTCCTCTGCGTTT
qPr-18s-F	ACAATTGGAGGGCAAGTCTG
qPr-18s-R	CACCGCGATAGGATTTTGAT

Table S3. Accession number of TRP channels identified in this study.

Gene name	Accession number
ZnTRPA1	XM_022083250.1
ZnPain-1	XM_022069106.1
ZnPain-2	XM_022072648.1
ZnPain-3	XM_022072708.1
ZnPain-4	XM_022065778.1
ZnPain-5	XM_022069064.1
ZnPain-6	XM_022069054.1
ZnPain-7	XM_022063669.1
ZnPyx	GenBank: KDR03815.1
ZnWtrw	XM_022057625.1
ZnNompC	XM_022059308.1
ZnIav	XR_002544018.1
ZnNan	XM_022086387.1
ZnTRPM	XM_022063775.1
ZnPkd2-1	XM_022062720.1
ZnPkd2-2	XM_022062967.1
ZnTRPML	XM_022068422.1
ZnTRP	XM_022057128.1
ZnTRPL	XM_022057105.1
ZnTRPγ	XM_022063395.1
ZnBrv2	XM_022087238.1
ZnTRPA5	XM_022085106.1
BgTRPA1	PSN57930.1
BgPain-1	PSN51663.1
BgPain-2	PSN47403.1
BgPain-3	PSN44088.1
BgPain-4	PSN50501.1
BgPain-5	PSN39524.1
BgPain - 6	PSN40730.1
BgPain-7	PSN28979.1
BgPyx	PSN45985.1
BgWtrw	PSN49738.1
BgNompC	PSN36722.1
BgIav	PSN51083.1
BgNan	PSN30164.1
BgTRPM	PSN39268.1
BgPkd2-1	PSN36947.1

BgPkd2-2	PSN29497.1
BgTRPML	PSN35272.1
BgTRP	PSN38237.1
BgTRPL	PSN38242.1
BgTRPγ	PSN33020.1
BgBrv3	PSN31034.1
BgTRPA5-1	PSN47462.1
BgTRPA5-2	PSN47911.1
NlTRPA1	XM_022335028.1
NlPain	XM_022340489.1
NlWtrw	XM_022345460.1
NlNompC	XM_022330016.1
NlIav	XM_022330203.1
NlNan	XM_022345641.1
NlTRPM	XM_022330672.1
NlPkd2	XM_022329028.1
NlTRPML	XM_022336950.1
NlTRP	XM_022338105.1
NlTRPL	XM_022338103.1
NlTRPγ	XM_022349498.1
DpTRPA1	OWR50481.1
DpPain	OWR52996.1
DpPyx	OWR52289.1
DpWtrw-1	OWR46419.1
DpWtrw-2	OWR45696.1
DpNompC	OWR50221.1
DpIav	OWR48059.1
DpNan	KZ248770.1
DpTRPM	OWR46589.1
DpTRPML	OWR43618.1
DpTRP	OWR42590.1
DpTRPL	OWR42591.1
DpTRPγ	OWR45522.1
DpTRPA5	OWR54184.1
MsTRPA1	XM_030164000.1
MsPain	XM_030167736.1
MsPyx	XM_030171035.1
MsWtrw-1	XM_030165492.1
MsWtrw-2	XM_030165499.1
MsNompC	XM_030165023.1

MsIav	XM_030175862.1
MsNan	XM_030173827.1
MsTRPM	XM_030174374.1
MsTRPML	XM_030178483.1
MsTRP	XM_030171785.1
MsTRPL	XM_030169886.1
MsTRPγ	XM_030177905.1
MsTRPA5-1	XM_030172372.1
MsTRPA5-2	XM_030172397.1
PxTRPA1	XM_013323649.1
PxPain	XM_013319882.1
PxPyx	XM_013315494.1
PxWtrw-1	XM_013310920.1
PxWtrw-2	XM_013310896.1
PxNompC	XM_013326120.1
PxIav	XM_013306586.1
PxNan	XM_013310965.1
PxTRPM	XM_013307087.1
PxTRPML	XM_013317138.1
PxTRP	XM_013316210.1
PxTRPL	XM_013316212.1
PxTRPγ	XM_013319481.1
PxTRPA5	XM_013327029.1
PrTRPA1	XM_022269136.1
PrPain	XM_022264961.1
PrPyx	XM_022260547.1
PrWtrw-1	XM_022263017.1
PrWtrw-2	XM_022263000.1
PrNompC	XM_022272967.1
PrIav	XM_022270177.1
PrNan	XM_022266197.1
PrTRPM	XM_022272780.1
PrTRPML	XM_022262542.1
PrTRP	XM_022260530.1
PrTRPL	XM_022259226.1
PrTRPγ	XM_022266966.1
PrTRPA5	XM_022269806.1
